# Supplementary material for: Analysis of 2.0 and 3.5 mm Cortical Bone Screw Dimensions
Source: Vet Sci. 2026 Jan 1;13(1):38. doi: 10.3390/vetsci13010038 (PMC12846576; doi:10.3390/vetsci13010038)
Supplement: Supplementary file 1 [file vetsci-13-00038-s001.zip › vetsci-4023299-supplementary.pdf]

Supplemental File S1: Raw Measurement Data of Individual Veterinary Bone Screws with Tolerance Status

Screw ID	Screw Size (mm)	Parameter	Location	Measured Value (mm)	Tolerance Status
**1**	2.0	Major Diameter	Proximal	1.950	In Tolerance
**1**	2.0	Major Diameter	Middle	2.000	In Tolerance
**1**	2.0	Major Diameter	Distal	2.000	In Tolerance
**1**	2.0	Pitch	Proximal	0.493	In Tolerance
**1**	2.0	Pitch	Middle	0.494	In Tolerance
**1**	2.0	Pitch	Distal	0.493	In Tolerance
**2**	2.0	Major Diameter	Proximal	2.078	Out of Tolerance
**2**	2.0	Major Diameter	Middle	2.065	In Tolerance
**2**	2.0	Major Diameter	Distal	2.052	In Tolerance
**2**	2.0	Pitch	Proximal	0.454	Out of Tolerance
**2**	2.0	Pitch	Middle	0.441	Out of Tolerance
**2**	2.0	Pitch	Distal	0.460	Out of Tolerance
**3**	2.0	Major Diameter	Proximal	2.079	Out of Tolerance
**3**	2.0	Major Diameter	Middle	2.104	Out of Tolerance
**3**	2.0	Major Diameter	Distal	2.072	Out of Tolerance
**3**	2.0	Pitch	Proximal	0.522	In Tolerance
**3**	2.0	Pitch	Middle	0.541	Out of Tolerance
**3**	2.0	Pitch	Distal	0.530	Out of Tolerance
**4**	2.0	Major Diameter	Proximal	2.029	In Tolerance
**4**	2.0	Major Diameter	Middle	2.030	In Tolerance
**4**	2.0	Major Diameter	Distal	2.036	In Tolerance
**4**	2.0	Pitch	Proximal	0.494	In Tolerance
**4**	2.0	Pitch	Middle	0.494	In Tolerance
**4**	2.0	Pitch	Distal	0.489	In Tolerance
**5**	2.0	Major Diameter	Proximal	2.076	Out of Tolerance
**5**	2.0	Major Diameter	Middle	2.079	Out of Tolerance
**5**	2.0	Major Diameter	Distal	2.099	Out of Tolerance
**5**	2.0	Pitch	Proximal	0.488	In Tolerance
**5**	2.0	Pitch	Middle	0.477	In Tolerance
**5**	2.0	Pitch	Distal	0.499	In Tolerance
**6**	2.0	Major Diameter	Proximal	1.970	In Tolerance
**6**	2.0	Major Diameter	Middle	1.990	In Tolerance
**6**	2.0	Major Diameter	Distal	2.060	In Tolerance
**6**	2.0	Pitch	Proximal	0.443	Out of Tolerance
**6**	2.0	Pitch	Middle	0.466	Out of Tolerance
**6**	2.0	Pitch	Distal	0.490	In Tolerance
**7**	2.0	Major Diameter	Proximal	1.949	In Tolerance
**7**	2.0	Major Diameter	Middle	1.951	In Tolerance
**7**	2.0	Major Diameter	Distal	1.952	In Tolerance
**7**	2.0	Pitch	Proximal	0.468	Out of Tolerance
**7**	2.0	Pitch	Middle	0.470	In Tolerance
**7**	2.0	Pitch	Distal	0.470	In Tolerance
**8**	2.0	Major Diameter	Proximal	2.000	In Tolerance
**8**	2.0	Major Diameter	Middle	1.924	In Tolerance
**8**	2.0	Major Diameter	Distal	1.974	In Tolerance
**8**	2.0	Pitch	Proximal	0.522	In Tolerance
**8**	2.0	Pitch	Middle	0.571	Out of Tolerance
**8**	2.0	Pitch	Distal	0.520	In Tolerance
**9**	2.0	Major Diameter	Proximal	1.976	In Tolerance
**9**	2.0	Major Diameter	Middle	1.980	In Tolerance
**9**	2.0	Major Diameter	Distal	1.977	In Tolerance
**9**	2.0	Pitch	Proximal	0.525	In Tolerance
**9**	2.0	Pitch	Middle	0.545	Out of Tolerance
**9**	2.0	Pitch	Distal	0.520	In Tolerance
**10**	2.0	Major Diameter	Proximal	1.974	In Tolerance
**10**	2.0	Major Diameter	Middle	1.948	In Tolerance
**10**	2.0	Major Diameter	Distal	2.000	In Tolerance
**10**	2.0	Pitch	Proximal	0.494	In Tolerance
**10**	2.0	Pitch	Middle	0.479	In Tolerance
**10**	2.0	Pitch	Distal	0.490	In Tolerance
**11**	2.0	Major Diameter	Proximal	2.090	Out of Tolerance
**11**	2.0	Major Diameter	Middle	2.120	Out of Tolerance
**11**	2.0	Major Diameter	Distal	2.000	In Tolerance
**11**	2.0	Pitch	Proximal	0.487	In Tolerance
**11**	2.0	Pitch	Middle	0.477	In Tolerance
**11**	2.0	Pitch	Distal	0.450	Out of Tolerance
**12**	2.0	Major Diameter	Proximal	2.080	Out of Tolerance
**12**	2.0	Major Diameter	Middle	2.050	In Tolerance
**12**	2.0	Major Diameter	Distal	1.990	In Tolerance
**12**	2.0	Pitch	Proximal	0.398	Out of Tolerance
**12**	2.0	Pitch	Middle	0.478	In Tolerance
**12**	2.0	Pitch	Distal	0.480	In Tolerance
**13**	2.0	Major Diameter	Proximal	2.086	Out of Tolerance
**13**	2.0	Major Diameter	Middle	2.004	In Tolerance

**13**	2.0	Major Diameter	Distal	2.001	In Tolerance
**13**	2.0	Pitch	Proximal	0.493	In Tolerance
**13**	2.0	Pitch	Middle	0.493	In Tolerance
**13**	2.0	Pitch	Distal	0.490	In Tolerance
**14**	2.0	Major Diameter	Proximal	2.003	In Tolerance
**14**	2.0	Major Diameter	Middle	2.004	In Tolerance
**14**	2.0	Major Diameter	Distal	2.057	In Tolerance
**14**	2.0	Pitch	Proximal	0.549	Out of Tolerance
**14**	2.0	Pitch	Middle	0.547	Out of Tolerance
**14**	2.0	Pitch	Distal	0.540	Out of Tolerance
**15**	2.0	Major Diameter	Proximal	2.056	In Tolerance
**15**	2.0	Major Diameter	Middle	2.026	In Tolerance
**15**	2.0	Major Diameter	Distal	1.956	In Tolerance
**15**	2.0	Pitch	Proximal	0.448	Out of Tolerance
**15**	2.0	Pitch	Middle	0.462	Out of Tolerance
**15**	2.0	Pitch	Distal	0.460	Out of Tolerance
**16**	2.0	Major Diameter	Proximal	1.948	In Tolerance
**16**	2.0	Major Diameter	Middle	1.949	In Tolerance
**16**	2.0	Major Diameter	Distal	1.950	In Tolerance
**16**	2.0	Pitch	Proximal	0.545	Out of Tolerance
**16**	2.0	Pitch	Middle	0.544	Out of Tolerance
**16**	2.0	Pitch	Distal	0.540	Out of Tolerance
**17**	2.0	Major Diameter	Proximal	2.077	Out of Tolerance
**17**	2.0	Major Diameter	Middle	2.188	Out of Tolerance
**17**	2.0	Major Diameter	Distal	2.122	Out of Tolerance
**17**	2.0	Pitch	Proximal	0.423	Out of Tolerance
**17**	2.0	Pitch	Middle	0.433	Out of Tolerance
**17**	2.0	Pitch	Distal	0.450	Out of Tolerance
**18**	2.0	Major Diameter	Proximal	1.998	In Tolerance
**18**	2.0	Major Diameter	Middle	2.011	In Tolerance
**18**	2.0	Major Diameter	Distal	2.113	Out of Tolerance
**18**	2.0	Pitch	Proximal	0.400	Out of Tolerance
**18**	2.0	Pitch	Middle	0.411	Out of Tolerance
**18**	2.0	Pitch	Distal	0.420	Out of Tolerance
**19**	2.0	Major Diameter	Proximal	1.974	In Tolerance
**19**	2.0	Major Diameter	Middle	1.974	In Tolerance
**19**	2.0	Major Diameter	Distal	1.982	In Tolerance
**19**	2.0	Pitch	Proximal	0.546	Out of Tolerance
**19**	2.0	Pitch	Middle	0.520	In Tolerance
**19**	2.0	Pitch	Distal	0.571	Out of Tolerance
**20**	2.0	Major Diameter	Proximal	1.974	In Tolerance
**20**	2.0	Major Diameter	Middle	1.952	In Tolerance
**20**	2.0	Major Diameter	Distal	2.000	In Tolerance
**20**	2.0	Pitch	Proximal	0.520	In Tolerance
**20**	2.0	Pitch	Middle	0.519	In Tolerance
**20**	2.0	Pitch	Distal	0.520	In Tolerance
**21**	2.0	Major Diameter	Proximal	2.079	Out of Tolerance
**21**	2.0	Major Diameter	Middle	1.949	In Tolerance
**21**	2.0	Major Diameter	Distal	2.026	In Tolerance
**21**	2.0	Pitch	Proximal	0.519	In Tolerance
**21**	2.0	Pitch	Middle	0.564	Out of Tolerance
**21**	2.0	Pitch	Distal	0.560	Out of Tolerance
**22**	2.0	Major Diameter	Proximal	2.079	Out of Tolerance
**22**	2.0	Major Diameter	Middle	2.052	In Tolerance
**22**	2.0	Major Diameter	Distal	2.080	Out of Tolerance
**22**	2.0	Pitch	Proximal	0.468	Out of Tolerance
**22**	2.0	Pitch	Middle	0.490	In Tolerance
**22**	2.0	Pitch	Distal	0.496	In Tolerance
**23**	2.0	Major Diameter	Proximal	1.949	In Tolerance
**23**	2.0	Major Diameter	Middle	1.951	In Tolerance
**23**	2.0	Major Diameter	Distal	1.952	In Tolerance
**23**	2.0	Pitch	Proximal	0.468	Out of Tolerance
**23**	2.0	Pitch	Middle	0.470	In Tolerance
**23**	2.0	Pitch	Distal	0.470	In Tolerance
**24**	2.0	Major Diameter	Proximal	2.000	In Tolerance
**24**	2.0	Major Diameter	Middle	1.924	In Tolerance
**24**	2.0	Major Diameter	Distal	1.974	In Tolerance
**24**	2.0	Pitch	Proximal	0.522	In Tolerance
**24**	2.0	Pitch	Middle	0.571	Out of Tolerance
**24**	2.0	Pitch	Distal	0.520	In Tolerance
**25**	2.0	Major Diameter	Proximal	1.976	In Tolerance
**25**	2.0	Major Diameter	Middle	1.980	In Tolerance
**25**	2.0	Major Diameter	Distal	1.978	In Tolerance
**25**	2.0	Pitch	Proximal	0.535	Out of Tolerance
**25**	2.0	Pitch	Middle	0.545	Out of Tolerance
**25**	2.0	Pitch	Distal	0.520	In Tolerance
**26**	2.0	Major Diameter	Proximal	1.974	In Tolerance
**26**	2.0	Major Diameter	Middle	1.948	In Tolerance

**26**	2.0	Major Diameter	Distal	2.000	In Tolerance
**26**	2.0	Pitch	Proximal	0.494	In Tolerance
**26**	2.0	Pitch	Middle	0.478	In Tolerance
**26**	2.0	Pitch	Distal	0.490	In Tolerance
**27**	2.0	Major Diameter	Proximal	2.090	Out of Tolerance
**27**	2.0	Major Diameter	Middle	2.120	Out of Tolerance
**27**	2.0	Major Diameter	Distal	2.010	In Tolerance
**27**	2.0	Pitch	Proximal	0.488	In Tolerance
**27**	2.0	Pitch	Middle	0.477	In Tolerance
**27**	2.0	Pitch	Distal	0.450	Out of Tolerance
**28**	2.0	Major Diameter	Proximal	2.080	Out of Tolerance
**28**	2.0	Major Diameter	Middle	2.060	In Tolerance
**28**	2.0	Major Diameter	Distal	1.990	In Tolerance
**28**	2.0	Pitch	Proximal	0.399	Out of Tolerance
**28**	2.0	Pitch	Middle	0.478	In Tolerance
**28**	2.0	Pitch	Distal	0.487	In Tolerance
**29**	2.0	Major Diameter	Proximal	2.086	Out of Tolerance
**29**	2.0	Major Diameter	Middle	2.005	In Tolerance
**29**	2.0	Major Diameter	Distal	2.001	In Tolerance
**29**	2.0	Pitch	Proximal	0.493	In Tolerance
**29**	2.0	Pitch	Middle	0.493	In Tolerance
**29**	2.0	Pitch	Distal	0.490	In Tolerance
**30**	2.0	Major Diameter	Proximal	2.003	In Tolerance
**30**	2.0	Major Diameter	Middle	2.014	In Tolerance
**30**	2.0	Major Diameter	Distal	2.057	In Tolerance
**30**	2.0	Pitch	Proximal	0.539	Out of Tolerance
**30**	2.0	Pitch	Middle	0.547	Out of Tolerance
**30**	2.0	Pitch	Distal	0.540	Out of Tolerance
**31**	2.0	Major Diameter	Proximal	2.003	In Tolerance
**31**	2.0	Major Diameter	Middle	2.000	In Tolerance
**31**	2.0	Major Diameter	Distal	2.000	In Tolerance
**31**	2.0	Pitch	Proximal	0.452	Out of Tolerance
**31**	2.0	Pitch	Middle	0.452	Out of Tolerance
**31**	2.0	Pitch	Distal	0.277	Out of Tolerance
**32**	2.0	Major Diameter	Proximal	2.032	In Tolerance
**32**	2.0	Major Diameter	Middle	2.	
